# Alterations of mitochondrial dynamics in serotonin transporter knockout rats: A possible role in the fear extinction recall mechanisms

**DOI:** 10.3389/fnbeh.2022.957702

**Published:** 2022-10-28

**Authors:** Paola Brivio, Maria Teresa Gallo, Peter Karel, Giulia Cogi, Fabio Fumagalli, Judith R. Homberg, Francesca Calabrese

**Affiliations:** ^1^Department of Pharmacological and Biomolecular Sciences, Università degli Studi di Milano, Milan, Italy; ^2^Department of Cognitive Neuroscience, Centre for Neuroscience, Donders Institute for Brain, Cognition and Behaviour, Radboud University Medical Center, Nijmegen, Netherlands

**Keywords:** stress-related disorders, 5-HTT^–/–^, mitochondria, fusion, fission

## Abstract

Stress-related mental disorders encompass a plethora of pathologies that share the exposure to a negative environment as trigger for their development. The vulnerability to the effects of a negative environment is not equal to all but differs between individuals based on the genetic background makeup. Here, to study the molecular mechanisms potentially underlying increased threat anticipation, we employed an animal model showing this symptom (5-HTT knockout rats) which we exposed to Pavlovian fear conditioning (FC). We investigated the role of mitochondria, taking advantage of the recent evidence showing that the dynamic of these organelles is dysregulated after stress exposure. Behavioral experiments revealed that, during the second day of extinction of the FC paradigm, 5-HTT knockout (5-HTT^–/–^) animals showed a lack of fear extinction recall. From a mechanistic standpoint, we carried out our molecular analyses on the amygdala and prefrontal cortex, given their role in the management of the fear response due to their tight connection. We demonstrated that mitochondrial dynamics are impaired in the amygdala and prefrontal cortex of 5-HTT^–/–^ rats. The dissection of the potential contributing factors revealed a critical role in the mechanisms regulating fission and fusion that are dysregulated in transgenic animals. Furthermore, mitochondrial oxidative phosphorylation, mitochondrial biogenesis, and the production of antioxidant enzymes were altered in these brain regions in 5-HTT^–/–^ rats. In summary, our data suggest that increased extracellular 5-HT levels cause an unbalance of mitochondrial functionality that could contribute to the reduced extinction recall of 5-HTT^–/–^ rats, pointing out the role of mitochondrial dynamics in the etiology of psychiatric disorders. Our findings, also, provide some interesting insights into the targeted development of drugs to treat such disorders.

## Introduction

Exposure to adverse life events is considered a key risk factor for the development of stress-related mental disorders, including depression, post-traumatic stress disorder, and anxiety (McEwen, 2012). Nevertheless, it has been widely recognized that such illnesses have a multifactorial etiology resulting from the interaction between adverse environmental stimuli and genetic factors (Caspi and Moffitt, 2006).

Unfortunately, despite the intense research in the field, to date, there is a limited understanding of the mechanisms underlying the increased vulnerability observed in the manifestation of these pathologies due to the gene x environment interactions, thus pushing the urgency to continue studying the systems responsible for their etiology, given their high societal costs.

Among the several gene loci investigated so far, the polymorphism of the promoter region of the gene (SLC64A) encoding for the serotonin transporter (5-HTT) has been associated with an increased vulnerability to stress-related disorders. Indeed, carriers of the 5-HTTLPR (5-HTT linked polymorphic region) short (S) allele are more sensitive to stressful events throughout life than long (L) carriers (Lesch et al., 1996; Caspi et al., 2010; Homberg and van den Hove, 2012).

To investigate, at the preclinical level, the role of 5-HTT in the disorders, 5-HTT knockout (5-HTT^–/–^) rats have been generated, modeling the 5-HTTLPR S-allele. These rats have been demonstrated to reproduce several of the phenotypes displayed by 5-HTTLPR s-allele carriers, including anxiety-like behavior, reduction in sociability, and heightened ambiguous threat perception (Homberg and Lesch, 2011; Schipper et al., 2019a,b). We previously demonstrated that 5-HTT^–/–^ rats and healthy 5-HTTLPR s-allele carriers show increased threat anticipation in the Pavlovian fear conditioning (FC) paradigm, which in both species was linked to altered functioning of a circuitry involving the prefrontal cortex, amygdala, and periaqueductal gray (Schipper et al., 2019a,b). Also, in patients with stress-related psychopathologies, threat anticipation measured using the FC paradigm is perturbed (Mott et al., 2012; Lancaster et al., 2016), making the readout attractive to further understand the underlying mechanisms.

**FIGURE 1:**
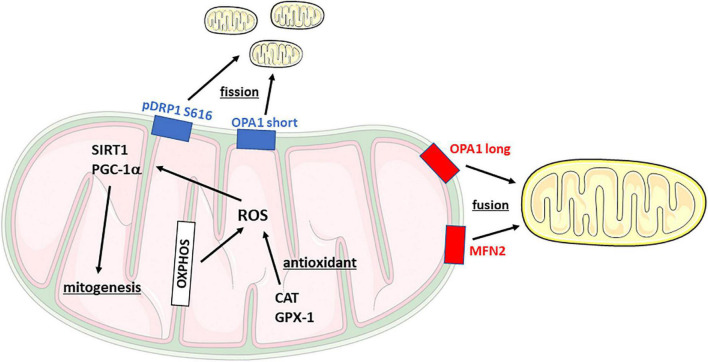
Schematic representation of mitochondrial dynamics and main functions. CAT, Catalase; DRP1, Dynamin-related protein 1; Gpx-1, Glutathione peroxidase 1; MFN2, Mitofusin 2; OPA1, Optic Atrophy Protein 1; OXPHOS, oxidative phosphorylation complex; PGC-1α, PPARG coactivator 1α; ROS, reactive oxygen species; SIRT1, Sirtuin 1.

In recent years, suboptimal mitochondrial function is emerging as a potential contributor to several psychiatric disorders (Shao et al., 2008; Preston et al., 2018) as a consequence of stress exposure (van der Kooij, 2020; Brivio et al., 2022). Accordingly, various factors implicated in mitochondrial functionality are dysregulated in patients affected by stress-related disorders (Su et al., 2008; MacDonald et al., 2019). Mitochondria are defined as the “power-house” of the cell since they provide the energy needed to satisfy all the cell requirements. Mitochondrial dynamics, the so-called fusion and fission machinery, play a fundamental role in the maintenance of functional mitochondria and the dynamism of these mechanisms occurs to guarantee the quality control of the cell. During fission, newly smaller mitochondria are generated to counteract negative external stimuli, whereas the process of fusion allows the elongation of mitochondrial structures and the transfer of the organelles’ content into newly fused mitochondria, a process activated to dilute cellular damage. The core regulatory proteins of these mechanisms are Dynamin-Related Protein 1 (DRP1) fission and Mitofusin 1-2 (MFN1-2) and Optic Atrophy protein 1 (OPA1) for fission and fusion, respectively, which are located on mitochondrial membranes (Figure 1). A continuous shift in fusion and fission mechanisms is needed to react to the external changes, such as metabolic and environmental stressors, but also to regulate synaptic plasticity and cognition (Picard and McEwen, 2014).

Thus, in an attempt to more comprehensively investigate the role of mitochondrial dynamics in the pathophysiology of stress-related disorders and to deepen the implication of serotonergic transmission on mitochondria (Fanibunda and Vaidya, 2021), the main goal of this study was to test 5-HTT^+/+^ and 5-HTT^–/–^ rats in a cued fear conditioning and extinction paradigm. We hypothesize that the reduced fear extinction response observed in transgenic animals could be due to impairments in metabolic pathways in the amygdala and prefrontal cortex caused by the genetic alteration itself and/or by a different activation exerted by FC exposure. The analyses were conducted in these brain regions because they are connected to functional circuitries involved in mechanisms of memory and learning, associated with the system that regulates fear extinction and directly implicated in fear-related mechanisms in both 5-HTT^–/–^ rats and 5-HTTLPR S-allele carriers (Duvarci and Pare, 2014; Giustino and Maren, 2015; Maren and Holmes, 2016; Schipper et al., 2019a,b).

## Materials and methods

### Animals

All experimental procedures were approved by the Central Committee on Animal Experiments (Centrale Commissie Dierproeven, CCD, The Hague, The Netherlands), and all efforts were made to minimize animal suffering and to reduce the number of animals used. 5-HTT^–/–^ rats (Slc6a41Hubr) were generated on a Wistar background by *N*-ethyl-*N*-nitrosurea (ENU)-induced mutagenesis (Smits et al., 2006) and were derived from crossing 5-HTT^+/–^ rats that were outcrossed for at least 12 generations with wild-type Wistar rats obtained from Harlan Laboratories (Horst, The Netherlands). Ear punches were taken at the age of 21 days for genotyping, which was done by Kbiosciences (Hoddesdon, United Kingdom). The animals used were derived from eight different litters, so some of the results showed may rely on data obtained from more than one rat from the same litter. All animals had *ad libitum* access to food and water. A 12-h light-dark cycle was maintained, with lights on at 08.00 a.m. All behavioral experiments were performed between 08.00 a.m. and 06:00 p.m.

### Study design, apparatus, and fear conditioning procedure

At postnatal day 70, half of 5-HTT^+/+^ and 5-HTT^–/–^ rats were randomly assigned to naïve and fear conditioning groups.

Fear conditioning was conducted in a 30.5 cm × 24.1 cm × 21 cm operant conditioning chamber (Model VFC-008, Med Associates, Sawbridgeworth Hertfordshire, United Kingdom). The box was housed within a sound-attenuating cubicle and contained a white LED stimulus light, a white and near-infrared house light, as well as a speaker capable of producing an 85 dB 2.8 kHz tone. The metal grid floor of the apparatus was connected to a scrambled shock generator (model ENV-412, Med Associates, Sawbridgeworth Hertfordshire, United Kingdom) that delivers shocks with an intensity of 0.6 mA intensity. Both the fear extinction and extinction recalls were conducted in a novel context, a 25 cm × 25 cm × 30 cm Plexiglas cage, with the bottom covered with a ±0.5 cm thick layer of black bedding, where 85 dB (measured at the center of the floor) 2.8 kHz auditory stimuli were delivered through a set of external speakers. The procedures were also conducted in a novel room.

**FIGURE 2:**
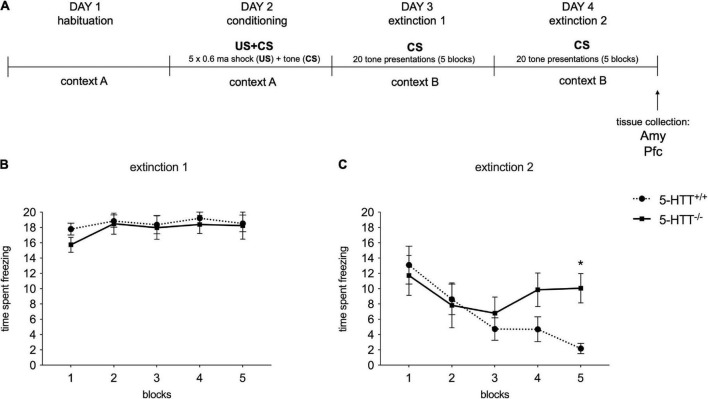
Fear conditioning behavioral results during the extinction recall sessions. **(A)** Schematic representation of the experimental paradigm. **(B,C)** The data are expressed as the mean of the time spent freezing during conditioned stimulus presentations ± SEM of 10 independent determinations. *p < 0.01 vs. 5-HTT^+/+^ (Two-way Anova with repeated measures). Amy, amygdala; Pfc, prefrontal cortex.

As depicted in Figure 2A, on postnatal day 70 animals were habituated to the conditioning context for 10 min. And 24 h after habituation, animals were given a cued fear conditioning session. Fear conditioning started with a 2-min habituation period, followed by five instances of a 30-s 85 dB 2.8 kHz auditory stimulus co-terminating with a 1-s 0.6 mA foot shock, followed by a 1-min inter-trial interval. And 24 and 48 h after conditioning, fear extinction and extinction recall were assessed. During extinction recall, rats were exposed to a 2-min habituation period, after which 20 presentations of 20 seconds of the auditory stimulus were given, with an inter-trial interval of 5 s. The freezing was automatically assessed by the Ethovision 9.0 behavioral software package (Noldus Information Technology B.V., Wageningen, the Netherlands) and was determined using the Activity Monitor feature of the software package, blind to the genotype of the animal. The apparatus was cleaned before and after each animal using a tissue slightly dampened with 70% EtOH.

To analyze fear extinction learning, extinction sessions were divided into five blocks representing the average freezing responses to four auditory cue presentations each. Average freezing to all auditory cue presentations during the recall sessions was used as an index for fear extinction recall.

Immediately at the end of the extinction recall procedure, animals were sacrificed through decapitation and the prefrontal cortex and amygdala were directly dissected from fresh tissues, frozen on dry ice, and stored at −80°C for molecular analyses.

Dissections were performed according to the atlas of Paxinos and Watson (2007). In detail, the prefrontal cortex (defined as Cg1, Cg3, and IL subregions corresponding to plates 6–10) was dissected from 2-mm thick slices, whereas the amygdala was dissected from the whole brain according to the plates 46–61 of the atlas.

### RNA preparation and gene expression analysis by quantitative real-time PCR

Total RNA was isolated by a single step of guanidinium isothiocyanate/phenol extraction using PureZol RNA isolation reagent (Bio-Rad Laboratories, Segrate, Italy) (Brivio et al., 2021). Real-time polymerase chain reaction (RT-PCR) was performed to assess *Cytochrome c oxidase 1* (*Cox1*) (Rn03296721_s1), *Cox3* (Rn03296820_s1), *Catalase* (*Cat*) (Rn00560930_m1), and *Glutathione peroxidase 1* (*Gpx1*) (Rn00577994_g1, Thermo Fisher Scientific, Monza, Italy) mRNA levels. RNA was analyzed by TaqMan qRT-PCR instrument (CFX384 real-time system, Bio-Rad Laboratories, Segrate, Italy) using the iScriptTM one-step RT-PCR kit for probes (Bio-Rad Laboratories, Italy). Samples were run in 384 well formats in triplicate as multiplexed reactions with the normalizing internal control *36B4* (primer fw TCAGTGCCTCACTCCATCAT, primer rev AGGAAGGCCTTGACCTTTTC, probe TGGATACAAAAGGGTCCTGG, Eurofins genomics, Vimodrone, Italy). A comparative cycle threshold (Ct) method was used to calculate the relative target gene expression.

### Protein extraction and western blot analysis

Western blot analysis was used to investigate phospho Dynamin-1-like protein (DRP1) Ser616, DRP1, mitofusin 2 (MFN2), Optic Atrophy Protein 1 (OPA1), oxidative phosphorylation (OXPHOS) complexes (C-II, C-IV subunit I, C-V alpha subunit), Sirtuin 1 (SIRT1), Peroxisome proliferator-activated receptor gamma coactivator 1-alpha (PGC1a), and CAT, in the whole homogenate. Tissues were manually homogenized using a glass-glass potter in a pH 7.4 cold buffer solution containing 150 mM NaCl, 50 mM Tris HCl, and 5 mM EDTA in the presence of a complete set of proteases (Roche, Monza, Italy), and phosphatase inhibitors (Merck Life Science S.r.l, Milano, Italy). The purity of the fraction obtained was detailed in Brivio et al. (2019). Total protein content was measured according to the Bradford Protein Assay procedure (Bio-Rad Laboratories S.r.l, Segrate, Italy), with the bovine serum albumin as a calibration standard. Equal amounts of protein were run under reducing conditions on 10% SDS-polyacrylamide gels and then electrophoretically transferred onto nitrocellulose membranes (Bio-Rad Laboratories S.r.l, Segrate, Italy). The blots were blocked with 5% non-fat dry milk (Euroclone, Milano, Italy), incubated with the primary antibodies, and then incubated with the opportune secondary antibody (see Table 1). Immunocomplexes were visualized by chemiluminescence using the Western Lightning Star ECL (Euroclone, Milano, Italy) and the Chemidoc MP imaging system (Bio-Rad Laboratories S.r.l, Segrate, Italy) (see Supplementary material for the whole blot images). Results were standardized using β-actin as the control protein, which was detected by evaluating the band density at 43 kDa.

**TABLE 1:** Antibodies conditions used in the western blot.

	Primary antibody	Secondary antibody
pDRP1 Ser616 (78–82 kDa)	1:1000 BSA 3% (Cell signaling), 4° O/N	Anti-rabbit 1:1000 BSA3%, 1h RT
DRP1 (78–82 kDa)	1:1000 BSA 3% (Cell signaling), 4° O/N	Anti-mouse 1:1000 BSA3%, 1h RT
OPA1 (80 kDa)	1:1000 BSA 3% (Cell signaling), 4° O/N	Anti-rabbit 1:1000 BSA3%, 1h RT
MFN2 (80 kDa)	1:1000 BSA 3% (Cell signaling), 4° O/N	Anti-rabbit 1:1000 BSA3%, 1h RT
OXPHOS (55–40–20 KDa)	1:2000 BSA 3% (Abcam), 4° O/N	Anti-mouse 1:2000 BSA3%, 1h RT
SIRT1 (120 Kda)	1:1000 BSA 5% (Cell signaling), 4° O/N	Anti-rabbit 1:1000 BSA5%, 1h RT
PGC1a (91 Kda)	1:1000 BSA 5% (Novus), 4° O/N	Anti-rabbit 1:1000 BSA5%, 1h RT
CAT (60 KDa)	1:1000 M3% (Cell signaling), 4° O/N	Anti-rabbit 1:1000 M3%, 1h RT
β-ACTIN (43 kDa)	1:10000 M3% (Sigma-Aldrich), 4° O/N	Anti-mouse 1:10000 M3%, 1h RT

BSA, Bovin serum albumin; M, Milk; O/N, overnight; RT, room temperature.

### Statistical analysis

**FIGURE 3:**
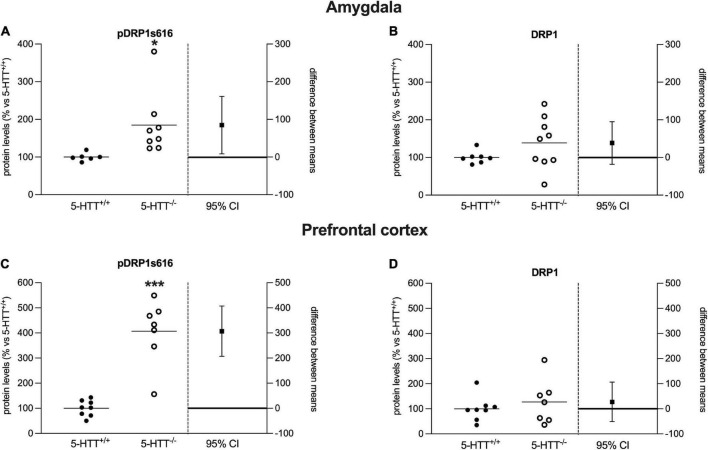
Analysis of DRP1 (phospho Ser616, total) protein levels in the amygdala **(A,B)** and in the prefrontal cortex **(C,D)** of 5-HTT^+/+^ and 5-HTT^–/–^ rats. The results are represented as Gardner–Altman plots. The black square shows the difference between the two means, and the error bar shows the 95% confidence interval of that difference. The data are expressed as a percentage of 5-HTT^+/+^ (set at 100%) of 6 to 10 independent determinations. *p < 0.05, ***p < 0.001 vs. 5-HTT^+/+^ (Unpaired *t*-test).

**FIGURE 4:**
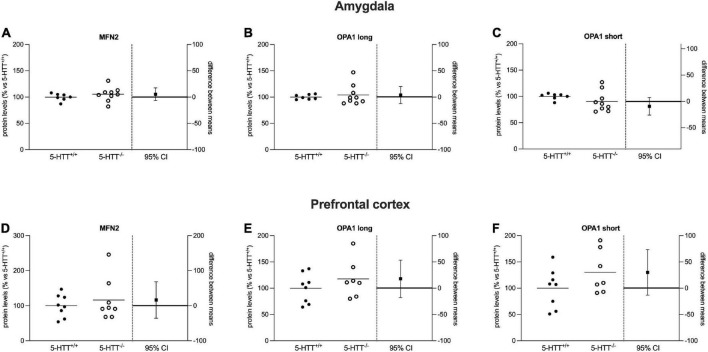
Analysis of MFN2 and OPA1 (long and short) protein levels in the amygdala **(A–C)** and in the prefrontal cortex **(D–F)** of 5-HTT^+/+^ and 5-HTT^–/–^ rats. The results are represented as Gardner–Altman plots. The black square shows the difference between the two means, and the error bar shows the 95% confidence interval of that difference. The data are expressed as a percentage of 5-HTT^+/+^ (set at 100%) of 6 to 10 independent determinations.

**FIGURE 5:**
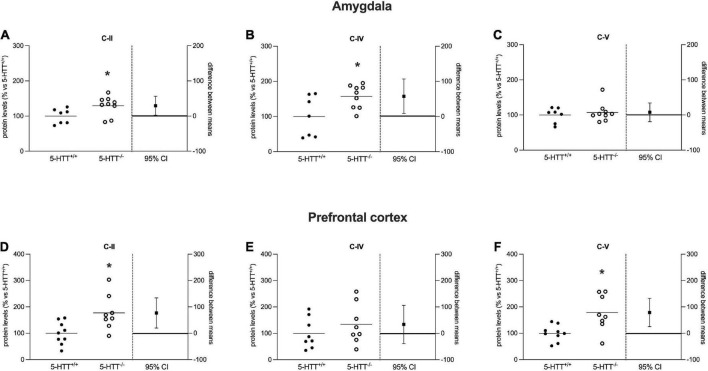
Analysis of OXPHOS complexes C-II, C-IV, and C-V protein levels in the amygdala **(A–C)** and in the prefrontal cortex **(D–F)** of 5-HTT^+/+^ and 5-HTT^–/–^ rats. The results are represented as Gardner–Altman plots. The black square shows the difference between the two means, and the error bar shows the 95% confidence interval of that difference. The data are expressed as a percentage of 5-HTT^+/+^ (set at 100%) of 6 to 10 independent determinations. *p < 0.05 vs. 5-HTT^+/+^ (Unpaired *t*-test).

**FIGURE 6:**
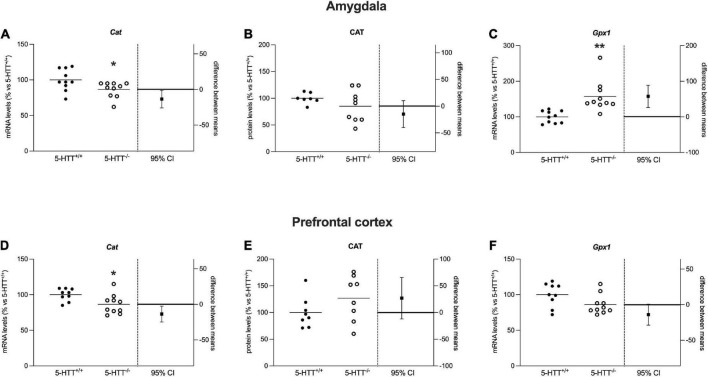
Analysis of Cat mRNA and protein levels and Gpx-1 mRNA levels in the amygdala **(A–C)** and in the prefrontal cortex **(D–F)** of 5-HTT^+/+^ and 5-HTT^–/–^ rats. The results are represented as Gardner–Altman plots. The black square shows the difference between the two means, and the error bar shows the 95% confidence interval of that difference. The data are expressed as a percentage of 5-HTT^+/+^ (set at 100%) of 6 to 10 independent determinations. *p < 0.05, **p < 0.01 vs. 5-HTT^+/+^ (Unpaired *t*-test).

**FIGURE 7:**
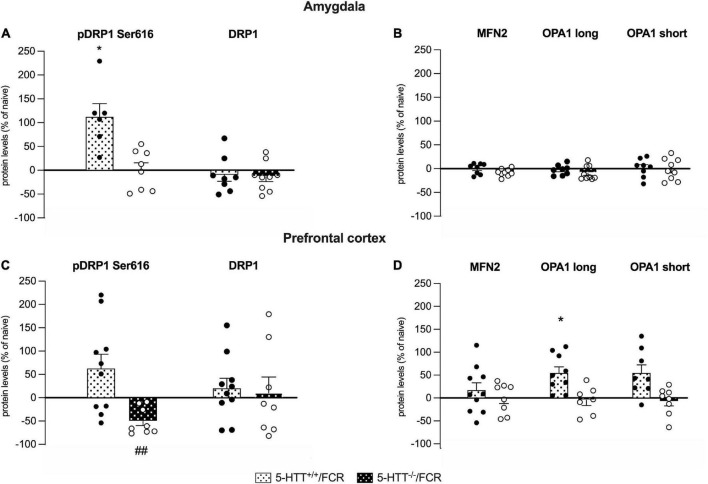
Analysis of the protein levels of the fission (pDRP1 Ser616 and total DRP1) and fusion machinery (MFN2, OPA1 long and short isoforms) in the amygdala **(A,B)** and in the prefrontal cortex **(C,D)** of 5-HTT^+/+^ and 5-HTT^–/–^ rats exposed to fear extinction recall (FCR). The data are expressed as a percentage of 5-HTT^+/+^/naive and 5-HTT^–/–^/naive and represent the mean ± SEM of 6 to 10 independent determinations. *p < 0.05 vs. 5-HTT^+/+^/naive; ##*p* < 0.01 vs. 5-HTT^–/*t*–^/naïve (Two-way Anova with Tukey).

**FIGURE 8:**
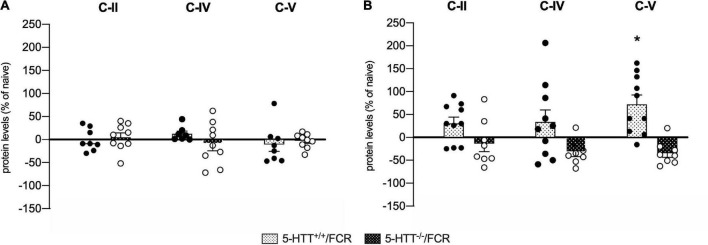
Analysis of OXPHOS complexes C-II, C-IV, and C-V protein levels in the amygdala **(A)** and in the prefrontal cortex **(B)** of 5-HTT^+/+^ and 5-HTT^–/–^ rats exposed to fear extinction recall (FCR). The data are expressed as a percentage of 5-HTT^+/+^/naive and 5-HTT^–/–^/naive and represent the mean ± SEM of 6 to 10 independent determinations. *p < 0.05 vs. 5-HTT^+/+^/naïve (Two-way Anova with Tukey).

**FIGURE 9:**
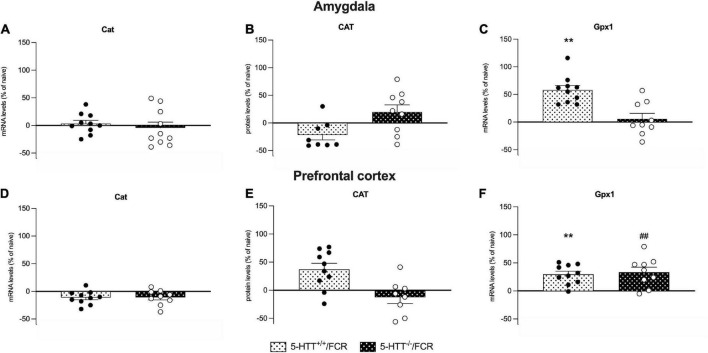
Analysis of Cat mRNA and protein levels and Gpx-1 mRNA levels in the amygdala **(A–C)** and prefrontal cortex **(D–F)** of 5-HTT^+/+^ and 5-HTT^–/–^ rats exposed to fear extinction recall (FCR). The data are expressed as a percentage of 5-HTT^+/+^/naive and 5-HTT^–/–^/naive and represent the mean ± SEM of 6 to 10 independent determinations. **p < 0.01 vs. 5-HTT^+/+^/naive; ^##^*p* < 0.01 vs. 5-HTT^–/–^/naïve (Two-way Anova with Tukey).

All statistical analyses were performed using SPSS Statistics version 24.0 (SPSS Inc., IBM, Armonk, NY, USA). Behavioral data were analyzed using a two-way analysis of variance (ANOVA) with repeated measures, followed by the Bonferroni *post-hoc* test, whereas the protein and gene expressions were analyzed using a two-way ANOVA with the Tukey *post-hoc* test or with the Unpaired *T*-test. Each experimental group consists of 10 rats. Significance for all tests was assumed for *p* < 0.05. Data are presented as Gardner–Altman plots (Figures 3–6) or as means ± standard error (SEM) (Figures 2, 7–9).

## Results

### 5-HTT^–/–^ rats show freezing behavior during the second fear extinction recall

We evaluated the behavioral effects of the fear conditioning protocol during the first (E1) and the second (E2) extinction recall session in 5-HTT^+/+^ and 5-HTT^–/–^ rats by measuring the time rats spent freezing after the exposure to conditioned stimuli in the five blocks.

In line with our previous evidence (Schipper et al., 2019b), we found no differences in the time spent freezing between genotypes in E1 (Figure 2B), while 5-HTT^–/–^ rats showed lower fear extinction recall than 5-HTT^+/+^ rats during E2 session, with 5-HTT^–/–^ rats freezing significantly more than their wild-type counterpart (+78%, *p* < 0.05 vs. 5-HTT^+/+^) during the 5th block of conditioned stimulus presentations (Figure 2C).

### Naïve 5-HTT^–/–^ rats show enhanced mitochondrial fission machinery

To investigate whether mitochondrial dynamics could be altered in rats lacking the 5-HTT, we measured the protein expression levels of the main marker of mitochondrial fission, DRP1, in the amygdala and prefrontal cortex of naïve 5-HTT^+/+^ and 5-HTT^–/–^ rats. In both brain regions, we found a significant increase in pDRP1 S616 (Amy: +85%, *p* < 0.05 vs. 5-HTT^+/+^; Pfc: +307%, *p* < 0.001 vs. 5-HTT^+/+^) in 5-HTT^–/–^ rats compared to 5-HTT^+/+^ rats (Figures 3A–C), whereas we did not observe any statistical difference between the two genotypes for the total form of DRP1 (Figures 3B–D).

### The fusion machinery is not altered in naïve 5-HTT^–/–^ rats

Regarding mitochondrial fusion, we focused on the major players of this mechanism, the MFN2 and OPA1 proteins.

As shown in Figure 4, we did not observe a significant effect of the genotype on the protein levels of MFN2 and OPA1 (long and short isoforms), either in the amygdala (Figures 4A–C) or in the prefrontal cortex (Figures 4D–F) of naïve 5-HTT^–/–^ and 5-HTT^+/+^ rats.

### Mitochondrial oxidative phosphorylation is altered in naive 5-HTT^–/–^ rats

Mitochondria generate ATP, the source of energy for the cell, through the mechanism of oxidative phosphorylation (OXPHOS) (Figure 1). The different OXPHOS complexes regulate the fine-tuning of bioenergetic control in response to external stimuli and alteration of OXPHOS-dependent energy production is implicated in the etiology of different diseases (Castellanos and Lanning, 2019).

Analyzing OXPHOS complexes protein levels in the amygdala, we observed a significant increase of C-II (+29%, *p* < 0.05 vs. 5-HTT^+/+^) and C-IV (+58%, *p* < 0.05 vs. 5-HTT^+/+^) in 5-HTT^–/–^ rats compared to 5-HTT^+/+^ rats (Figures 5A,B), whereas we did not observe a modulation for the C-V (Figure 5C).

As shown in Figures 5D–F, in the prefrontal cortex, we found a significant upregulation of C-II (+77%, *p* < 0.05 vs. 5-HTT^+/+^) and C-V (+79%, *p* < 0.01 vs. 5-HTT^+/+^) protein expression in 5-HTT^–/–^ rats relative to 5-HTT^+/+^ rats, but no changes for C-IV.

### *Cox3* mRNA levels are enhanced in the prefrontal cortex of naïve 5-HTT^–/–^ rats

We next measured the gene expression of *Cox-1* and *Cox-3*, the catalytic subunits of cytochrome c oxidase, the last enzyme in the respiratory electron transport chain, in the amygdala, and in the prefrontal cortex (Tables 2A,B). In the amygdala, we did not observe any modulation due to the genotype, either for *Cox-1* or *Cox-3* mRNA levels (Table 2A).

**TABLE 2:** Analysis of Cox-1 and Cox-3 mRNA levels, SIRT1 and PGC-1α protein levels in the amygdala (A,C) and in the prefrontal cortex (B,D) of 5-HTT^+/+^ and 5-HTT^–/–^ rats.

Gene	Amygdala (A)		Prefrontal cortex (B)	
		
	5-HTT^+/+^	5-HTT^–/–^	5-HTT^+/+^	5-HTT^–/–^
Cox-1	100 ± 9	128 ± 11	100 ± 13	132 ± 11
Cox-3	100 ± 5	104 ± 5	100 ± 4	197 ± 20***

**Protein**	**Amygdala (C)**		**Prefrontal cortex (D)**	
		
	**5-HTT^+/+^**	**5-HTT^–/–^**	**5-HTT^+/+^**	**5-HTT^–/–^**

SIRT1	100 ± 4	198 ± 27**	100 ± 21	152 ± 8*
PGC-1α	100 ± 7	119 ± 12	100 ± 3	109 ± 14

The data are expressed as a percentage of 5-HTT^+/+^ (set at 100%) and represent the mean ± SEM of 7 to 10 independent determinations. *p < 0.05, **p < 0.01, ***p < 0.001 vs. 5-HTT^+/+^ (Unpaired *t*-test).

By contrast, as shown in Table 2B, in the prefrontal cortex, we found a statistically significant upregulation of *Cox-3* (+97%, *p* < 0.001 vs. 5-HTT^+/+^) mRNA levels in 5-HTT^–/–^ rats in comparison to 5-HTT^+/+^, while we did not observe any difference between the two genotypes for *Cox-1* mRNA levels.

### Naïve 5-HTT^–/–^ rats show increased SIRT1 protein levels in the amygdala and prefrontal cortex

We then focused on the protein expression of SIRT1 and PGC-1α, two master regulators of mitochondrial biogenesis and function (Hock and Kralli, 2009; Wareski et al., 2009; Figure 1). In the amygdala (Table 2C), we observed an upregulation of SIRT1 protein levels in 5-HTT^–/–^ rats compared to 5-HTT^+/+^ rats (+98%, *p* < 0.01 vs. 5-HTT^+/+^), while we did not find any difference for PGC-1α between the two genotypes. Similarly, in the prefrontal cortex, we observed an increased expression of SIRT1 (+52%, *p* < 0.05 vs. 5-HTT^+/+^) in 5-HTT^+/+^ vs. 5-HTT^–/–^ with no changes for PGC-1α protein levels (Table 2D).

### Antioxidant mediators are differently modulated in naïve 5-HTT^–/–^ rats

During oxidative phosphorylation, mitochondria generate a significant proportion of reactive oxygen species (ROS) whereas antioxidant systems and enzymes are activated to balance the concentration of ROS. As antioxidant enzymes, we measured the gene and protein expression of Catalase and Glutathione Peroxidase 1 (Figure 1).

As shown in Figure 6A, in the amygdala, we observed a significant reduction of *Cat* mRNA levels (−13%, *p* < 0.05 vs. 5-HTT^+/+^) in 5-HTT^–/–^ rats with respect to their 5-HTT^+/+^ counterpart, while we did not observe any statistically significant modulation for CAT protein levels (Figure 6B). Moreover, we found that 5-HTT^–/–^ rats showed a significant increase of *Gpx1* gene expression in comparison to 5-HTT^+/+^ (+57%, *p* < 0.01 vs. 5-HTT^+/+^) (Figure 6C).

Similarly, in the prefrontal cortex, we found a significant downregulation of *Cat* mRNA levels (−13%, *p* < 0.05 vs. 5-HTT^+/+^) in 5-HTT^–/–^ compared to 5-HTT^+/+^ rats (Figure 6D) and no changes for the protein levels among the two genotypes (Figure 6E). In this brain region, we did not observe any statistically significant alteration of *Gpx-1* gene expression (Figure 6F).

### Fear extinction recall enhances fission and fusion machinery, respectively, in the amygdala and prefrontal cortex of 5-HTT^+/+^ rats

Given the crucial role of mitochondrial dynamics for neuronal plasticity as well as for memory formation and recall (Messina et al., 2020), we studied the possible involvement of the markers mentioned above in the deficits observed in the fear extinction recall.

As shown in Figure 7A, in the amygdala, we observed a significant increase of pDRP1 S616 specifically in 5-HTT^+/+^ but not in 5-HTT^–/–^ rats after fear extinction recall (+112%, *p* < 0.05 vs. 5-HTT^+/+^/naive), whereas we did not observe any modulation of total DRP1 levels.

Differently, in the prefrontal cortex, we found a significant downregulation of pDRP1 S616 after fear extinction recall, specifically in 5-HTT^–/–^ rats (−49%; *p* < 0.01 vs. in 5-HTT^–/–^/naive), while we did not observe any effect of fear conditioning recall on total DRP1 protein levels (Figure 7C).

Regarding the fusion machinery (Figure 7D), we observed a significant enhancement of OPA1 long (+55%, *p* < 0.05 vs. 5-HTT^+/+^/naive) and a trend of increase of OPA1 short (+55%, *p* > 0.05 vs. 5-HTT^+/+^/naive) protein levels in association with the fear extinction recall in the prefrontal cortex of 5-HTT^+/+^animals, an effect we did not find in the same brain region of 5-HTT^–/–^ rats (Figure 7D). We did not observe any modulation of MFN2 in both genotypes.

In the amygdala, we did not find any modulation in association with fear extinction recall, either in 5-HTT^+/+^ or in 5-HTT^–/–^ rats, for both MFN2 and OPA1 (long and short isoforms) (Figure 7B).

### Fear extinction recall enhances OXPHOS C-V protein expression in the prefrontal cortex of 5-HTT^+/+^ rats

In the amygdala (Figure 8A), we did not observe any effects in association with fear extinction recall, neither in 5-HTT^+/+^ nor in 5-HTT^–/–^ rats for the three oxidative phosphorylation complexes investigated. In the prefrontal cortex, on the other hand, we found that exposure to fear extinction recall increased C-V protein levels specifically in 5-HTT^+/+^ rats (+72%, *p* < 0.05 vs. 5-HTT^+/+^/naive) (Figure 8B).

### *Cox-3* mRNA levels are increased in association with fear extinction recall in the prefrontal cortex of 5-HTT^+/+^ rats

As shown in Table 3B, we found an upregulation of *Cox-3* mRNA levels (+61%, *p* < 0.05 vs. 5-HTT^+/+^/naive) specifically in the prefrontal cortex of 5-HTT^+/+^ rats exposed to fear extinction recall, compared to the naive counterpart (Table 3B). On the contrary, no effects were found in the amygdala (Table 3A).

**TABLE 3:** Analysis of Cox-1 and Cox-3 mRNA levels, SIRT1 and PGC-1α protein levels in the amygdala (A,C) and in the prefrontal cortex (B,D) of 5-HTT^+/+^ and 5-HTT^–/–^ rats exposed to fear extinction recall (FCR).

Gene	Amygdala (A)		Prefrontal cortex (B)	
		
	Δ5-HTT^+/+^/FCR	Δ5-HTT^–/–^/FCR	Δ5-HTT^+/+^/FCR	Δ5-HTT^–/–^/FCR
Cox-1	+39% ± 17	+5% ± 9	−18% ± 17	+34% ± 22
Cox-3	+14% ± 8	+15% ± 8	+61% ± 21*	−14% ± 6

**Protein**	**Amygdala (C)**		**Prefrontal cortex (D)**	
		
	Δ**5-HTT^+/+^/FCR**	Δ**5-HTT**^–/–^**/FCR**	Δ**5-HTT^+/+^/FCR**	Δ**5-HTT**^–/–^**/FCR**

SIRT1	+21% ± 12	+14% ± 16	+87% ± 39*	−38 ± 10
PGC-1α	+6% ± 12	−5% ± 10	+32% ± 6	+5 ± 12

The data are expressed as a percentage of 5-HTT^+/+^/naive and 5-HTT^–/–^/naive and represent the mean ± SEM of 6–10 independent determinations. *p < 0.05 vs. 5-HTT^+/+^/naïve (Two-way Anova with Tukey).

### SIRT1 protein levels are upregulated in association with fear extinction recall in the prefrontal cortex of 5-HTT^+/+^ rats

We then considered protein expression of mitochondrial biogenesis regulators SIRT1 and PGC-1α in the amygdala and prefrontal cortex of 5-HTT^+/+^ and 5-HTT^–/–^ rats after they experienced a 4-day fear conditioning and extinction protocol.

In the amygdala, we did not find significant effects on SIRT1 and PGC-1α protein expression due to fear extinction recall both in 5-HTT^+/+^ and in 5-HTT^–/–^ rats (Table 3C).

By contrast, as shown in Table 3D, in the prefrontal cortex, we observed that fear extinction recall induced an increase in SIRT1 (+87%, *p* < 0.05 vs. 5-HTT^+/+^/naive) levels in 5-HTT^+/+^ animals, suggesting an increase in mitochondrial biogenesis during the activation of fear extinction.

### Fear extinction recall increases *Gpx1* expression in the amygdala and the prefrontal cortex

*Gpx1* mRNA levels was upregulated after the fear extinction recall in the amygdala of 5-HTT^+/+^ (+58%, *p* < 0.01 vs. 5-HTT^+/+^/naive) but not in 5-HTT^–/–^ rats (Figure 9C), while in the prefrontal cortex, fear extinction recall was associated with a significant upregulation of *Gpx1* mRNA levels both in 5-HTT^+/+^ (+30%, *p* < 0.01 vs. 5-HTT^+/+^/naive) and in 5-HTT^–/–^ (+34%, *p* < 0.001 vs. 5-HTT^–/–^/naive) rats, compared to their naive counterpart (Figure 9F).

We did not observe any significant effect of fear extinction recall on catalase mRNA and protein levels in both brain regions (Figures 9A,B,D,E) either in 5-HTT^+/+^ or in 5-HTT^–/–^ rats.

## Discussion

In this study, we confirm previous observations that 5-HTT^–/–^ rats showed an impairment of the fear extinction recall (Narayanan et al., 2011; Shan et al., 2014; Schipper et al., 2019a,b), a behavioral response that is typically observed in patients affected by stress-related disorders (Lissek et al., 2005; Shin and Liberzon, 2010; Norrholm et al., 2011; Cover et al., 2014). Notably, here we present for the first time that such impairment is associated with alterations of mitochondrial dynamics in the amygdala and prefrontal cortex.

In line with our previous evidence (Schipper et al., 2019a,b), we observed that 5-HTT^–/–^ rats showed a significantly increased freezing behavior during the extinction period highlighting the validity of this model as a tool to investigate the mechanisms underlying the impairment of threat perception.

Since alterations of mitochondrial functions have been related to psychiatric disorders at both clinical and preclinical levels (Shao et al., 2008; Daniels et al., 2020), we hypothesized that the behavior observed in 5-HTT^–/–^ rats could be due, at least in part, to an imbalance of mitochondrial activity related to the congenital lack of the 5-HTT. Indeed, it has been speculated that serotonin exerts antioxidant-like effects and may modulate mitochondrial energy production (Azmitia, 2007; Fanibunda and Vaidya, 2021). Moreover, accumulating evidence shows that alterations of the serotonergic system can cause mitochondrial dysfunctions, thus leading to the manifestation of psychiatric disorders (Muller et al., 2016; Hollis et al., 2017; Villa et al., 2017).

Mitochondrial dynamics act as a regulator of brain function and cognition as the energy that mitochondria provide is fundamental for the maintenance of synaptic plasticity. Indeed, alteration of energy levels can impair the transmission of the information at the synaptic level with a negative impact on memory and learning mechanisms, despite the process of memory engravement and recall not being clear (Picard and McEwen, 2014).

When examining the main marker of fission, DRP1, we observed increased phosphorylation of DRP1 in S616 specifically in 5-HTT^–/–^ animals, both in the amygdala and prefrontal cortex. Moreover, fear extinction recall, in wild-type rats, increased the phosphorylation of DRP1 S616 in the amygdala and presumably also in the prefrontal cortex, where we observed a tendency toward a similar increase albeit not significant. Conversely, rats lacking the 5-HTT were unable to exhibit such enhancement and even in the prefrontal cortex, a significant decrease of DRP1 phosphorylation is observed. These data suggest that mitochondrial fission contributes to extinction recall and that the underlying mechanisms controlling such behavior are dysregulated in these brain areas of knockout animals.

The same reasoning can be applied when examining the long and short isoforms of OPA1. Also for this protein, knockout rats were unable to increase the expression of such isoforms, at variance from wild-type animals. However, this critical mediator of mitochondrial fusion is upregulated only in the prefrontal cortex. Thus, it appears that mitochondrial fusion contributes to extinction recall as well, primarily at the cortical level. It is important to note that Li et al. (2019) showed that increased levels of OPA1 and fusion were connected to improved memory and cognition in mice. Moreover, alterations in the expression of genes associated with mitochondrial function were found in the amygdala of an animal model of PTSD (Li et al., 2014). These results together reveal the importance of proper mitochondrial dynamics for the maintenance of correct brain functions.

When analyzing the main actors of mitochondrial oxidative phosphorylation, we observed that the deletion of 5-HTT was associated with the upregulation of OXPHOS C-II—C-IV complexes in the amygdala and OXPHOS C-II—C-V complexes in the prefrontal cortex. Such alterations in OXPHOS levels may be connected to misfunctions in the oxidative phosphorylation process, in accordance with a study reporting that a pharmacologically induced 5-HTT inhibition damaged the activity of the supercomplexes (Li et al., 2012). Furthermore, patients with major depression showed an increase in OXPHOS complex levels and an alteration of ATP production in the brain (Martins-de-Souza et al., 2012). The defects we observed in oxidative phosphorylation would be critical, not only leading to ATP depletion and energy shortage for cells but also enhancing the production of free radicals, physiologically generated by this process and not properly counteracted. In addition, it appears that fear extinction recall relies on the enhancement of OXPHOS complex V levels as shown by wild-type animals, whose expression was instead significantly downregulated in the prefrontal cortex of the transgenic rats.

In line with the OXPHOS dysregulation, we found that 5-HTT^–/–^ animals showed a significant decrease in antioxidant enzyme *Cat* mRNA levels in the amygdala and prefrontal cortex and increased expression of *Gpx1* in the amygdala, possibly indicating an alteration of the activity of these enzymes to cope with the possible increase of ROS, due to the OXPHOS alteration. Similarly Alzoubi et al. (2019) observed a reduction in CAT activity in an animal model for post-traumatic stress disorder.

Further, *Cox-3* was upregulated in the prefrontal cortex in 5-HTT^+/+^ rats exposed to fear extinction recall. This corresponds to the finding that a reduction in Cox activity was paralleled by neural damage and deficits in memory and learning in rat brains (de la Torre et al., 1997). The findings are also matching those showing that the activity of Cox in the amygdala in a rat model of post-traumatic stress disorder was associated with behavioral abnormalities (Xiao et al., 2011). Taken together, the data suggest that an increase in Cox expression is important for the promotion of fear extinction recall.

Finally, looking at SIRT1, a master regulator of mitochondrial biogenesis and function (Hock and Kralli, 2009; Wareski et al., 2009), we observed an increase of its protein levels in 5-HTT^–/–^ rats both in the amygdala and prefrontal cortex. In addition, we found that SIRT1 was increased in the prefrontal cortex of 5-HTT^+/+^ animals that show fear extinction recall, effects that were not present in the same brain region of 5-HTT^–/–^ rats. This suggests that the cortical enhancement of the SIRT1—PGC-1α axis might be enrolled to meet cellular energy demand and stimulate the mechanisms of memory and learning that may sustain the extinction response. The deletion of SIRT1 has been linked to defects in synaptic plasticity and impaired memory (Michán et al., 2010) and, in line with our findings, Wahl et al. (2019) demonstrated that SIRT1 expression is required for the fear extinction in mice.

Our study is associated with some limitations that require attention. First of all, since we used only male rats, the findings here described cannot be generalized to female subjects. Second, the molecular analyses were carried out at a single time point, that is, at the end of the second day of extinction, and therefore we cannot draw any conclusions on the moment in which such changes were established. In addition, further analyses, and more targeted technological approaches, such as oxygen respirometry and cell type discrimination, are needed to strengthen the causality of our data.

In conclusion, in this study, we found that genetic ablation of the serotonin transporter is associated with a pattern of dysregulations of mitochondrial targets in the amygdala and prefrontal cortex, two areas of the brain known to be involved in mechanisms of memory and learning as well as in processing threat sensitivity (Pezawas et al., 2005). We propose that mitochondrial dysregulation could be the basis of the fear extinction recall deficit of 5-HTT^–/–^ rats, deserving further investigation in future research.

## Data availability statement

The original contributions presented in this study are included in the article/Supplementary material, further inquiries can be directed to the corresponding author.

## Ethics statement

The animal study was reviewed and approved by Centrale Commissie Dierproeven (CCD), The Hague, Netherlands.

## Author contributions

PK performed the experiment and collected the behavioral data. PB, MG, and GC performed and analyzed the molecular data. PB analyzed the data and wrote the manuscript. JH and FC designed the study and interpreted the data. JH, FF, and FC reviewed and edited the manuscript. All authors critically reviewed the content and approved the final version for publication.
