# Supplementary material for: Alterations of mitochondrial dynamics in serotonin transporter knockout rats: A possible role in the fear extinction recall mechanisms
Source: Front Behav Neurosci. 2022 Oct 28;16:957702. doi: 10.3389/fnbeh.2022.957702 (PMC9650094; doi:10.3389/fnbeh.2022.957702)
Supplement: Supplementary file 1 [file Data_Sheet_1.PDF]

Amy

Experimental groups:

$5HTT^{+/+}$ / naïve : 3-10 — $5HTT^{-/-}$ / naïve : 31-40

$5HTT^{+/+}$ / FCR: 61-69 — $5HTT^{-/-}$ / FCR: 91-100

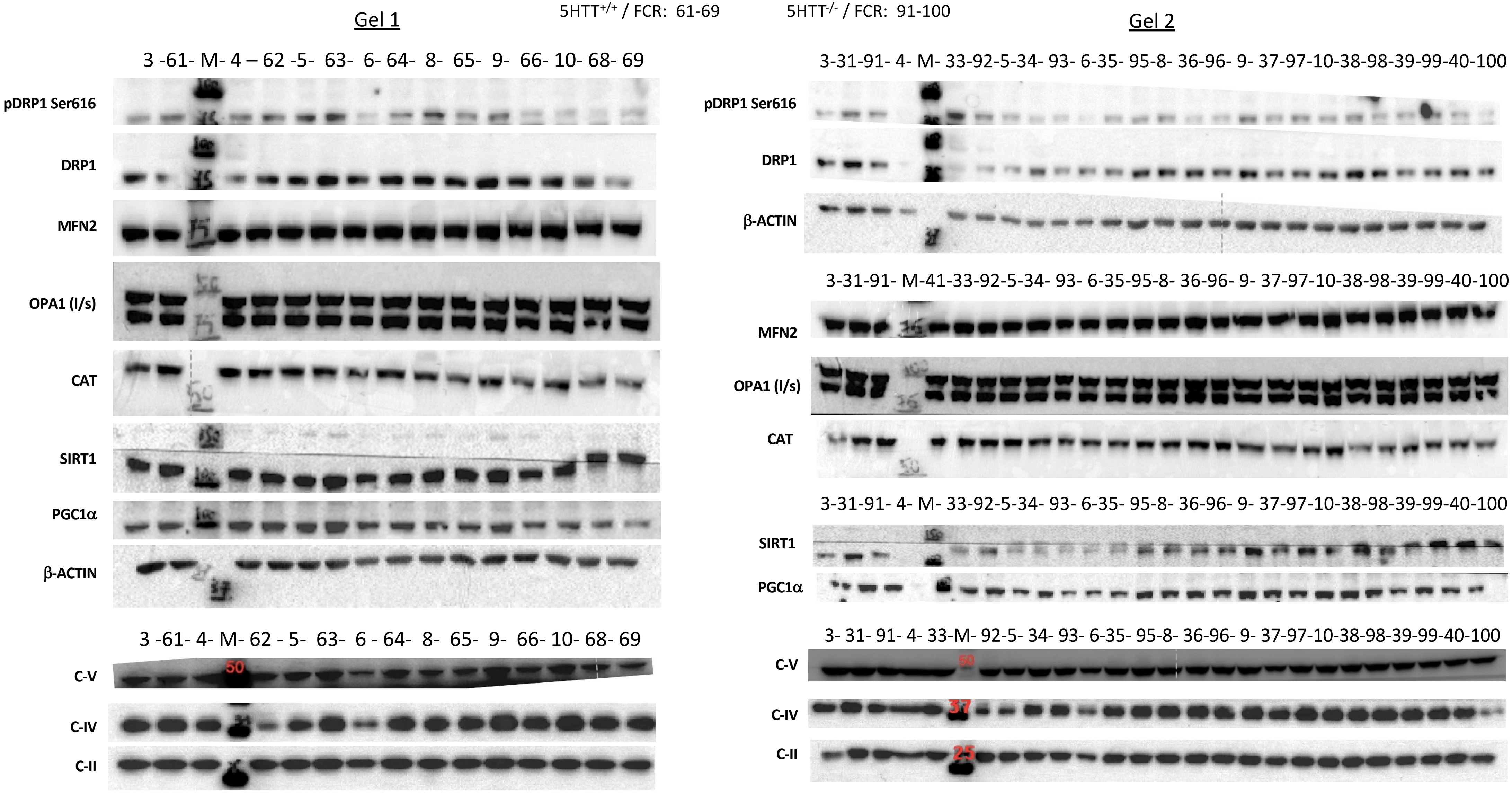

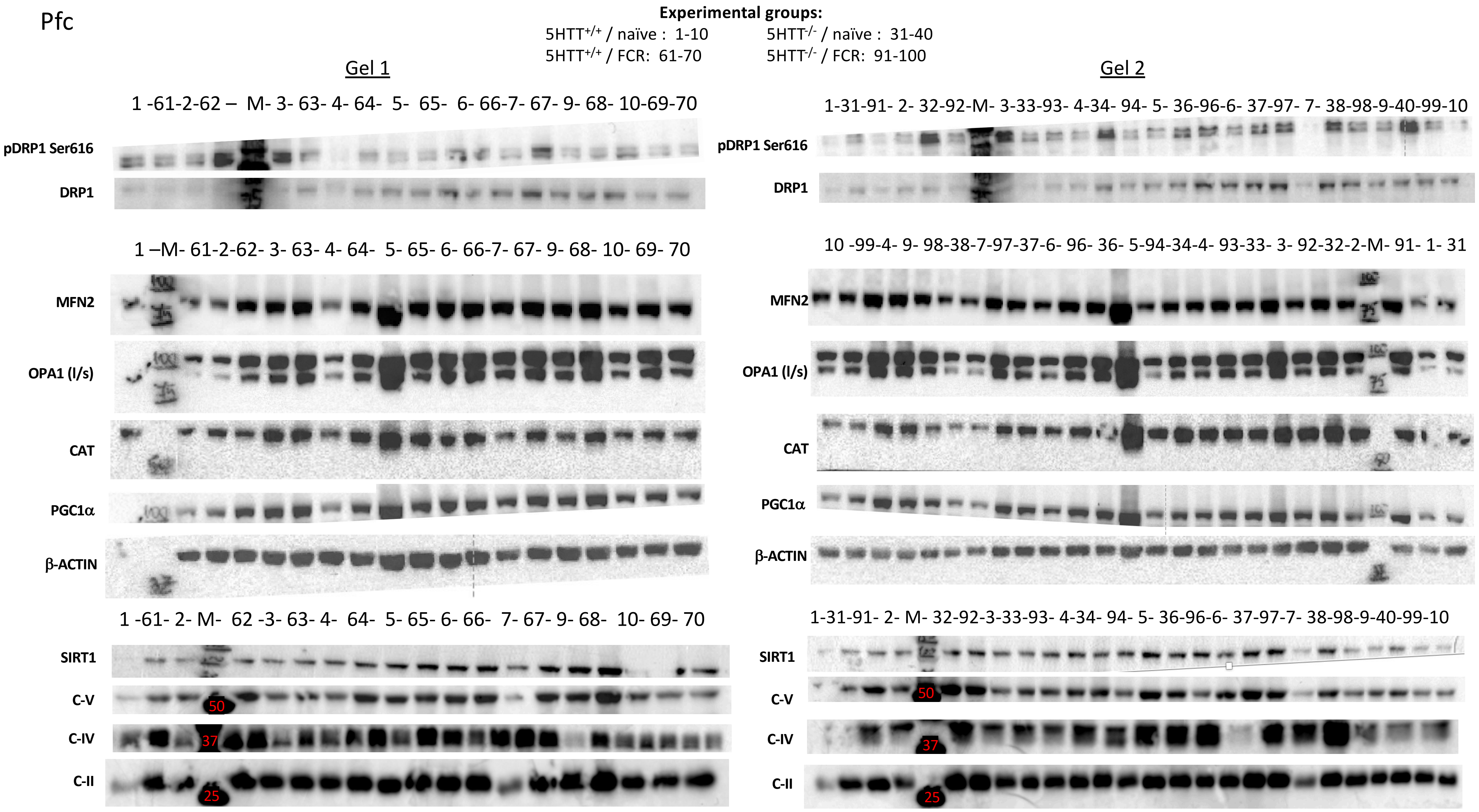

Pfc
Experimental groups:
5HTT+/+ / naïve : 1-10
5HTT-/- / naïve : 31-40
5HTT+/+ / FCR: 61-70
5HTT-/- / FCR: 91-100
Gel 1
Gel 2
1 -61-2-62 – M- 3- 63- 4- 64- 5- 65- 6- 66-7- 67- 9- 68- 10-69-70
1-31-91- 2- 32-92-M- 3-33-93- 4-34- 94- 5- 36-96-6- 37-97- 7- 38-98-9-40-99-10
pDRP1 Ser616
DRP1
1 –M- 61-2-62- 3- 63- 4- 64- 5- 65- 6- 66-7- 67- 9- 68- 10- 69- 70
10 -99-4- 9- 98-38-7-97-37-6- 96- 36- 5-94-34-4- 93-33- 3- 92-32-2-M- 91- 1- 31
MFN2
OPA1 (l/s)
CAT
PGC1α
β-ACTIN
1 -61- 2- M- 62 -3- 63- 4- 64- 5- 65- 6- 66- 7- 67- 9- 68- 10- 69- 70
1-31-91- 2- M- 32-92-3-33-93- 4-34- 94- 5- 36-96-6- 37-97-7- 38-98-9-40-99-10
SIRT1
C-V
C-IV
C-II
50
37
25
