# Supplementary material for: Alterations of mitochondrial dynamics in serotonin transporter knockout rats: A possible role in the fear extinction recall mechanisms
Source: Front Behav Neurosci. 2022 Oct 28;16:957702. doi: 10.3389/fnbeh.2022.957702 (PMC9650094; doi:10.3389/fnbeh.2022.957702)
Supplement: Supplementary file 2 [file Data_Sheet_2.docx]

Supplementary Material

# Supplementary tables

| **Behaviour** | | | |
| --- | --- | --- | --- |
|  | Two-way ANOVA | F (dFn, dFd) | P value |
| ***Extinction day 2*** | Genotype | F (1,19) = 0.731 | p = 0.4039 |
|  | Time | F (5,15) = 11.4 | p = 0.000 |
|  | Genotype X Time | F (5,15) = 3.51 | p = 0.0061 |

**Supplementary table 1: Two-way ANOVA analysis with repeated measures of behavioral data of 5-HTT^+/+^ and 5-HTT^-/-^  rats after the second extinction recall session**

| **Amygdala** | | | | |  |  |  |
| --- | --- | --- | --- | --- | --- | --- | --- |
| **Proteins** | **Two-way ANOVA** | **F (dFn, dFd)** | **P value** | |  |  |  |
| **pDRP1s616** | **Genotype** | F (1,27) = 1.254 | p = 0.274 | |  |  |  |
|  | **FC** | F (1,27) = 4.661 | p = 0.041 | |  |  |  |
|  | **Genotype X FC** | F (1,27) = 4.379 | p = 0.047 | |  |  |  |
| **DRP1** | **Genotype** | F (1,32) = 4.341 | p = 0.046 | | |  |  |
|  | **FC** | F (1,32) = 0.731 | p = 0.400 | | |  |  |
|  | **Genotype X FC** | F (1,32) = 0.074 | p = 0.788 | | |  |  |
| **MFN2** | **Genotype** | F (1,32) = 0.087 | p = 0.770 | | |  |  |
|  | **FC** | F (1,32) = 1.441 | p = 0.240 | | |  |  |
|  | **Genotype X FC** | F (1,32) = 1.452 | p = 0.238 | | |  |  |
| **OPA1 Long** | **Genotype** | F (1,32) = 0.011 | | p = 0.917 | | | |
|  | **FC** | F (1,32) = 1.566 | | p = 0.221 | | | |
|  | **Genotype X FC** | F (1,32) = 0.449 | | p = 0.508 | | | |
| **OPA1 Short** | **Genotype** | F (1,32) = 2.779 | | p = 0.106 | | | |
|  | **FC** | F (1,32) = 0.003 | | p = 0.953 | | | |
|  | **Genotype X FC** | F (1,32) = 0.034 | | p = 0.856 | | | |
| **OXPHOS CII** | **Genotype** | F (1,32) = 10.625 | | p = 0.003 | | | |
|  | **FC** | F (1,32) = 0.081 | | p = 0.778 | | | |
|  | **Genotype X FC** | F (1,32) = 0.112 | | p = 0.741 | | | |
| **OXPHOS CIV** | **Genotype** | F (1,31) = 6.009 | | p = 0.021 | | |  |
|  | **FC** | F (1,31) = 0.002 | | p = 0.961 | | |  |
|  | **Genotype X FC** | F (1,31) = 0.555 | | p = 0.462 | | |  |
| **OXPHOS CV** | **Genotype** | F (1,32) = 1.312 | | p = 0.261 | | |  |
|  | **FC** | F (1,32) = 0.506 | | p = 0.483 | | |  |
|  | **Genotype X FC** | F (1,32) = 0.138 | | p = 0.713 | | |  |
| ***Cox-1*** | **Genotype** | F (1,37) = 1.456 | | p = 0.236 | | |  |
|  | **FC** | F (1,37) = 4.541 | | p = 0.040 | | |  |
|  | **Genotype X FC** | F (1,37) = 1.070 | | p = 0.308 | | |  |
| ***Cox-3*** | **Genotype** | F (1,38) = 0.656 | p = 0.423 | |  |  |  |
|  | **FC** | F (1,38) = 4.776 | p = 0.036 | |  |  |  |
|  | **Genotype X FC** | F (1,38) = 0.048 | p = 0.828 | |  |  |  |
| **SIRT1** | **Genotype** | F (1,32) = 18.113 | p = 0.000 | |  |  |  |
|  | **FC** | F (1,32) = 1.043 | p = 0.316 | |  |  |  |
|  | **Genotype X FC** | F (1,32) = 0.022 | p = 0.884 | |  |  |  |
| **PGC-1α** | **Genotype** | F (1,32) = 1.282 | p = 0.267 | |  |  |  |
|  | **FC** | F (1,32) = 0.004 | p = 0.951 | |  |  |  |
|  | **Genotype X FC** | F (1,32) = 0.272 | p = 0.606 | |  |  |  |
| ***Cat*** | **Genotype** | F (1,38) = 11.598 | p = 0.002 | | |  |  |
|  | **FC** | F (1,38) = 0.249 | p = 0.621 | | |  |  |
|  | **Genotype X FC** | F (1,38) = 1.156 | p = 0.290 | | |  |  |
| **CAT** | **Genotype** | F (1,32) = 0.239 | p = 0.629 | | |  |  |
|  | **FC** | F (1,32) = 0.064 | p = 0.802 | | |  |  |
|  | **Genotype X FC** | F (1,32) = 4.245 | p = 0.048 | | |  |  |
| ***Gpx1*** | **Genotype** | F (1,37) = 6.909 | | p = 0.013 | | | |
|  | **FC** | F (1,37) = 7.305 | | p = 0.011 | | | |
|  | **Genotype X FC** | F (1,37) = 7.616 | | p = 0.009 | | | |
| **Supplementary table 2: Two-way ANOVA analysis of protein and mRNA levels in the amygdala of 5-HTT^+/+^ and 5-HTT^-/-^ rats exposed to fear conditioning.** | | | | |  |  |  |
| **Prefrontal cortex** | | | | |  |  |  |
| **Proteins** | **Two-way ANOVA** | **F (dFn, dFd)** | **P value** | |  |  |  |
| **pDRP1s616** | Genotype | F (1,32) = 25.153 | p = 0.000 | |  |  |  |
|  | FC | F (1,32) = 3.952 | p = 0.056 | |  |  |  |
|  | Genotype X FC | F (1,32) = 14.283 | p = 0.000 | |  |  |  |
| **DRP1** | Genotype | F (1,33) = 1.425 | p = 0.242 | | |  |  |
|  | FC | F (1,33) = 0.005 | p = 0.944 | | |  |  |
|  | Genotype X FC | F (1,33) = 0.313 | p = 0.580 | | |  |  |
| **MFN2** | Genotype | F (1,33) = 0.222 | p = 0.641 | | |  |  |
|  | FC | F (1,33) = 0.260 | p = 0.614 | | |  |  |
|  | Genotype X FC | F (1,33) = 0.284 | p = 0.598 | | |  |  |
| **OPA1 Long** | Genotype | F (1,30) = 0.964 | | p = 0.335 | | | |
|  | FC | F (1,30) = 3.593 | | p = 0.069 | | | |
|  | Genotype X FC | F (1,30) = 5.642 | | p = 0.025 | | | |
| **OPA1 Short** | Genotype | F (1,30) = 0.008 | | p = 0.929 | | | |
|  | FC | F (1,30) = 2.294 | | p = 0.142 | | | |
|  | Genotype X FC | F (1,30) = 4.314 | | p = 0.047 | | | |
| **OXPHOS CII** | Genotype | F (1,34) = 5.685 | | p = 0.023 | | | |
|  | FC | F (1, 34) = 0.026 | | p = 0.873 | | | |
|  | Genotype X FC | F (1, 34) = 1.665 | | p = 0.207 | | | |
| **OXPHOS CIV** | Genotype | F (1, 33) = 0.042 | | p = 0.839 | | | |
|  | FC | F (1, 33) = 0.045 | | p = 0.833 | | | |
|  | Genotype X FC | F (1, 33) = 2.779 | | p = 0.106 | | | |
| **OXPHOS CV** | Genotype | F (1,34) = 0.410 | | p = 0.527 | | | |
|  | FC | F (1,34) = 0.093 | | p = 0.762 | | | |
|  | Genotype X FC | F (1,34) = 13.034 | | p = 0.001 | | | |
| ***Cox-1*** | Genotype | F (1,36) = 11.690 | p = 0.002 | |  |  |  |
|  | FC | F (1,36) = 0.495 | p = 0.487 | |  |  |  |
|  | Genotype X FC | F (1,36) = 2.873 | p = 0.099 | |  |  |  |
| ***Cox-3*** | Genotype | F (1,37) = 10.955 | p = 0.002 | |  |  |  |
|  | FC | F (1,37) = 1.085 | p = 0.305 | |  |  |  |
|  | Genotype X FC | F (1,37) = 7.381 | p = 0.010 | |  |  |  |
| **SIRT1** | Genotype | F (1,34) = 0.651 | | p = 0.426 | | |  |
|  | FC | F (1,34) = 0.310 | | p = 0.582 | | |  |
|  | Genotype X FC | F (1,34) = 7.859 | | p = 0.009 | | |  |
| **PGC-1α** | Genotype | F (1,32) = 0.253 | | p = 0.619 | | |  |
|  | FC | F (1,32) = 3.578 | | p = 0.069 | | |  |
|  | Genotype X FC | F (1,32) = 1.901 | | p = 0.179 | | |  |
| ***Cat*** | Genotype | F (1,37) = 10.392 | | p = 0.003 | | |  |
|  | FC | F (1,37) = 7.318 | | p = 0.011 | | |  |
|  | Genotype X FC | F (1,37) = 0.062 | | p = 0.805 | | |  |
| **CAT** | Genotype | F (1,33) = 0.000 | | p = 0.987 | | |  |
|  | FC | F (1,33) = 0.702 | | p = 0.409 | | |  |
|  | Genotype X FC | F (1,33) = 4.487 | | p = 0.043 | | |  |
| ***Gpx1*** | Genotype | F (1,37) = 6.048 | | p = 0.019 | | |  |
|  | FC | F (1,37) = 25.771 | | p = 0.000 | | |  |
|  | Genotype X FC | F (1,37) = 0.003 | | p = 0.959 | | |  |

**Supplementary table 3: Two-way ANOVA analysis of protein and mRNA levels in the prefrontal cortex of 5-HTT^+/+^ and 5-HTT^-/-^ rats exposed to fear conditioning.**
